# Crystal structures of zinc(II) com­plexes with β-hy­droxy­pyri­dine­carboxyl­ate ligands: examples of structure-directing effects used in inorganic crystal engineering

**DOI:** 10.1107/S2052520621000299

**Published:** 2021-02-27

**Authors:** Nóra Veronika May, Kevin Nys, H. Y. Vincent Ching, Laura Bereczki, Tamás Holczbauer, Valerio B. Di Marco, Petra Bombicz

**Affiliations:** a Centre for Structural Sciences, Research Centre for Natural Sciences, Magyar tudósok körútja 2, Budapest, H-1117, Hungary; b Department of Chemistry, University of Antwerp, Universiteitsplein 1, Antwerpen, B-2610, Belgium; c Institute of Materials and Environmental Chemistry, Research Centre for Natural Sciences, Magyar tudósok körútja 2, Budapest, H-1117, Hungary; d Institute of Organic Chemistry, Research Centre for Natural Sciences, Magyar tudósok körútja 2, Budapest, H-1117, Hungary; e Department of Chemical Sciences, University of Padova, via Marzolo 1, Padova, 35131, Italy

**Keywords:** zinc(II) com­plex, hydroxypyri­dinecarboxylic acid, inorganic crystal engineering, trigonal bipyramidal geometry, tau value, CSD, coordination geometry

## Abstract

The coordination properties of four hy­droxy­pyri­dine­carboxyl­ates, designed for the treatment of iron-overloading con­ditions as bidentate *O*,*O*′-donor ligands, have been studied with Zn^II^ in the solid state. The coordination com­pounds [Zn(A1)_2_(H_2_O)_2_] (**1**), [Zn(A2)_2_(H_2_O)] (**2**), [Zn(A3)_2_(H_2_O)]·2H_2_O (**3**) and [Zn_2_(B1)_4_(H_2_O)_2_]·4H_2_O (**4**), where the ligands are 1-methyl-4-oxidopyridinium-3-­carboxyl­ate (A1, C_7_H_6_NO_3_), 1,6-di­methyl-4-oxidopyridinium-3-carboxyl­ate (A2, C_8_H_8_NO_3_), 1,5-di­methyl-4-oxido-pyridinium-3-carboxyl­ate (A3, C_8_H_8_NO_3_) and 1-methyl-3-oxidopyridinium-4-carboxyl­ate (B1, C_7_H_6_NO_3_), have been synthesized and analysed by single-crystal X-ray diffraction. The ligands were chosen to probe (i) the electronic effects of inverting the positions of the O-atom donor groups (*i.e.* A1 *versus* B1) and (ii) the electronic and steric effects of the addition of a second methyl group in different positions on the pyri­dine ring. Two axially coordinated water molecules resulting in a six-coordinated symmetrical octahedron com­plement the bis-ligand com­plex of A1. Ligands A2 and A3 form five-coordinated trigonal bipyramidal com­plexes with one additional water molecule in the coordination sphere, which is a rarely reported geometry for Zn^II^ com­plexes. Ligand B1 shows a dimeric structure, where the two Zn^2+^ dications have slightly distorted octahedral geometry and the pyridino­late O atom of the neighbouring com­plex bridges them. The coordination spheres of the Zn^2+^ dications and the supramolecular structures are discussed in detail. The packing arrangements of **1**–**3** are similar, having alternating hydro­philic and hydro­phobic layers, however the similarity is broken in **4**. The obtained coordination geometries are com­pared with their previously determined Cu^II^ analogues. The study of the individual com­plexes is com­plemented with a com­prehensive analysis of Zn^II^ com­plexes with oxygen donor ligands with data from the Cambridge Structural Database.

## Abstract 2

The structure-directing effects of four *O*,*O*′-donor hy­droxy­pyri­dine­carboxyl­ate derivatives in their com­plexation with Zn^II^ have been investigated by single-crystal X-ray diffraction. One octahedral and two trigonal bipyramidal mononuclear com­plexes, as well as a pyridino­late-bridged dinuclear complex with octahedral geometry, were detected.

## 1. Introduction   

Hy­droxy­pyri­dine­carb­oxy­lic acid (HPC) derivatives have been considered (Di Marco *et al.*, 2002 ▸; Crisponi *et al.*, 2013 ▸; Sija *et al.*, 2014 ▸; Dean *et al.*, 2018 ▸) as potential chelating agents for the treatment of iron-overloading con­ditions. The design of these com­pounds was based on deferiprone (1,2-di­methyl-3-hy­droxy­pyridin-4-one), which is a globally used iron-chelating drug. *In vitro* studies have shown that Cu^II^ and Zn^II^ are the most com­petitive metal ions against Fe^III^ and are able to considerably affect the formation of Fe^III^ com­plexes of these iron chelators (Clarke & Martell, 1992 ▸; Pashadilis & Kontoghiorghes, 2001 ▸; Li, 2019 ▸). Investigating the com­plexation of HPCs to Cu^II^ and Zn^II^ is of importance as the displacement of these essential metal ions by chelating drugs could adversely affect the biological processes dependent on these metals, potentially causing toxicity. The com­plexation properties of several HPCs towards Cu^II^ in the solid and solution states have been reported recently (May *et al.*, 2019 ▸), showing the influence of electron distribution on the coordination properties of Cu^II^ with HPCs of different methyl, hy­droxy­ethyl and carb­oxy­ethyl derivatives. While all HPC ligands were found to coordinate Cu^II^ through the deprotonated O-atom donors (oxide and carboxylate), their arrangement in [Cu*L*_2_] resulted in distinct structures. Solution equilibrium studies and density functional theory (DFT) calculations revealed a significant difference in the electronegativity of the donor carboxyl­ate and hydroxy O atoms. A correlation between the increasing acidity of the OH group with com­plex stability was observed. These electronic differences can also be used to rationalize the formation of bridging dimers, as well as of *cis* or *trans* arrangements. Solution speciation of ligand A3 (DQ715, 1,5-di­methyl-4-oxidopyridinium-3-carboxyl­ate) with Cu^II^ and Zn^II^ has been reported previously (Sija *et al.*, 2014 ▸) and it was found that A3 forms only mononuclear com­plexes with Zn^II^, *i.e.* [Zn*L*] and [Zn*L*_2_], and the stabilities of the formed com­plexes are lower com­pared to their Cu^II^ analogues. With these divalent metal ions the stability of the obtained com­plex is significantly lower than the stability with Fe^III^ or Al^III^, which makes HPCs good candidates as Fe^III^ or Al^III^ chelators.

**Figure 1:**
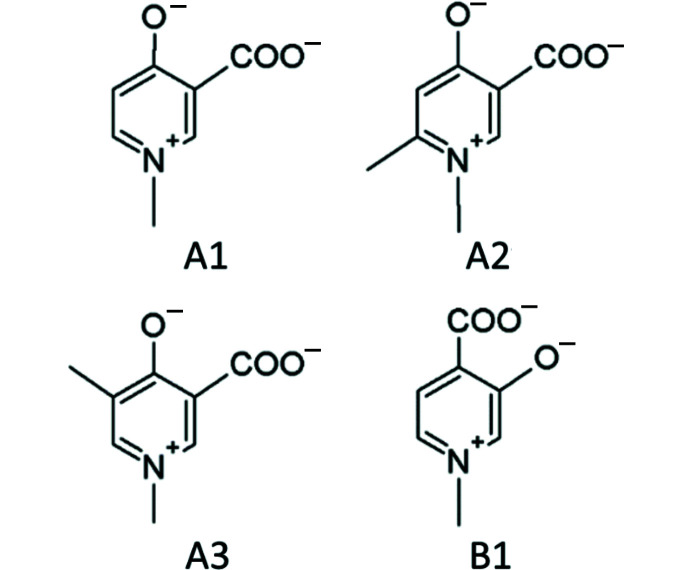
The structures of the investigated ligands in their fully deprotonated ionic forms. From now on, the com­pounds in this article will be referred to with the names A1–A3 and B1, omitting the charge for the sake of simplicity. In the literature relating to these com­pounds, the following acronyms have been used for A1–A3 and B1, respectively: DQ1, DQ716, DQ715 and DT1 (Crisponi *et al.*, 2013 ▸).

Following on from this previous work, we report here our solid-state studies on the com­plexation to Zn^II^ of four HPC ligands, namely, 1-methyl-4-oxidopyridinium-3-­carboxyl­ate (A1), 1,6-di­methyl-4-oxidopyridinium-3-carboxyl­ate (A2), 1,5-di­methyl-4-oxido-pyridinium-3-carboxyl­ate (A3) and 1-methyl-3-oxidopyridinium-4-carboxyl­ate (B1) (Fig. 1 ▸), by single-crystal X-ray diffraction. The ligands were selected to investigate the different effects on com­plexation of (i) the inductive effects when the positions of the O-atom donor groups are inverted (*i.e.* A1 *versus* B1) and (ii) the electronic and steric effects of the addition of a second methyl group in different positions on the pyri­dine ring (*i.e.* A1 *versus* A2 *versus* A3). We have also com­pared the structures of the Zn^II^ com­plexes of A1–A3 and B1 with other previously reported O-atom-donor ligand-containing Zn^II^ com­plexes. A com­prehensive coordination geometry analysis by data mining using the Cambridge Structural Database (CSD, Version 2020.1; Groom *et al.*, 2016 ▸) was performed. When available, the Zn^II^ com­plex structures of A1–A3 and B1 were also com­pared with their Cu^II^ analogues (Figs. S1–S3 in the supporting information) because although Cu^II^ and Zn^II^ can form com­plexes with the same ligands, the geometries of the resulting com­plexes are usually different owing to their different electronic configurations. As Cu^II^ has a *d* ^9^ electronic configuration, the most common geometries are elongated octahedral or square pyramidal, as a consequence of the Jahn–Teller effect, while in the case of Zn^II^, the *d* ^10^ electronic configuration prefers the very symmetrical tetragonal and octahedral geometries.

Our structural com­parison of Cu^II^ and Zn^II^ com­plexes containing the same and related ligands also revealed the structural features originating from (i) the steric and electronic effects of the ligands themselves, (ii) the geometrical preferences of the metal ions and (iii) the intermolecular forces between the molecules in the crystals. These are important aspects of the goals of inorganic crystal engineering (ICE), where coordination bonds connect metal ions and organic building blocks to each other. ICE aims at a better understanding of structure-directing effects in order to find strategies to control molecular self-assembly (Biradha *et al.*, 2011 ▸; Desiraju, 2003 ▸).

## 2. Experimental   

### 2.1. Chemicals and crystallization   

HPCs were synthesized as described previously (Di Marco *et al.*, 2006 ▸; Dean *et al.*, 2009 ▸, 2014 ▸). The Zn^II^ stock solution was prepared from ZnCl_2_ (Sigma–Aldrich) dissolved in doubly distilled water. The concentration was checked by ICP–OES. The NaOH, HCl and buffer solutions used in the pH adjustment were purchased from Sigma–Aldrich. Typically, the ligand (2 mg) was dissolved in water (2 ml) and ZnCl_2_ solution was added to obtain a twofold ligand excess. The pH was adjusted with NaOH to 7.0. After about 2–3 weeks, colourless single crystals appeared. Single crystals suitable for X-ray diffraction were obtained by slow evaporation from aqueous solution. A crystal was selected for the diffraction experiment from among the several crystals which were left in the saturated solution in order to preserve their quality (yields were *ca* 50%).

### 2.2. X-ray data collection, structure solution and refinement of com­pounds 1–4   

Crystal data, data collection and structure refinement details for **1**–**4** are summarized in Table 1 ▸. H atoms were placed in geometric positions and were included in structure-factor calculations. In general, C-bound H atoms were geometrically located and refined as riding (assuming distances of C—H = 0.96 Å for methyl and C—H = 0.93 Å for aromatic protons, and were refined by *U*_iso_ = 1.5*U*_eq_ for methyl and *U*_iso_ = 1.2*U*_eq_ for aromatic carrier atoms. The water H atoms were located from difference Fourier maps and then the positions of H_2_O were refined as rigid units. Selected bond lengths and angles were calculated using *PLATON* software (Spek, 2020 ▸). The calculated powder X-ray diffraction (PXRD) patterns were generated from the single-crystal X-ray diffraction data using *PLATON* (see Figs. S4–S7 in the supporting information).

### 2.3. Hirshfeld surface analysis   

The Hirshfeld surfaces of the investigated molecules in the crystals of **1**–**4** were calculated by *CrystalExplorer* (Turner *et al.*, 2017 ▸; Spackman & Jayatilaka, 2009 ▸; Spackman & McKinnon, 2002 ▸; McKinnon *et al.*, 2004 ▸). High-resolution Hirshfeld surfaces were mapped with the functions *d*_norm_ (normalized contact distance). The Hirshfeld surface of a molecule is generated by points where the contribution to the electron density from the molecule of interest is equal to the contribution from all neighbouring molecules. Each point of this surface has two distances: *d*_e_ is the distance from the point to the nearest nucleus external to the surface and *d*_i_ the distance to the nearest nucleus internal to the surface. The combination of *d*_e_ and *d*_i_ in the form of a two-dimensional (2D) fingerprint plot results in unique properties for each crystal and provides a useful tool to com­pare the intermolecular contacts in the different crystals. Distances involving H atoms were normalized in all calculations of the Hirshfeld surfaces (the C—H and O—H distances were 1.083 and 0.983 Å, respectively). The atomic distances given in the tables and figures throughout this article were calculated based on the single-crystal X-ray diffraction measurements.

## 3. Results and discussions   

### 3.1. Proton dissociation processes of the ligands   

The deprotonation steps of the ligands (AH_2_^+^) have been determined previously and it was found that the first proton dissociation at very low pH (p*K*_a1_ < 1) can be assigned to the –COOH group. In the neutral AH forms, the –OH proton is involved in an intramolecular hydrogen bond with the deprotonated –COO^−^ group (Fig. S8 in the supporting information). The fully deprotonated A^−^ form can be obtained by the second deprotonation at the –OH group which is therefore accom­panied by the cleavage of this internal hydrogen bond. The p*K*_a2_ values are influenced by the inductive effect of the positively charged >N^+^—Me groups and the other ring substituents. Another influencing factor is keto–enol tautomerization, which is more likely to occur for 4-hy­droxypyri­dine-3-carboxyl­ates (A1–A3) than for 3-hy­droxypyri­dine-4-carboxyl­ates (B1) (Fig. S8). The previously determined p*K*_a2_ values resulted in the deprotonation order A1 [5.9578 (6)] < A2 [6.295 (1)] < B1 [6.6326 (8)] < A3 [6.64 (1)] (Di Marco *et al.*, 2009 ▸; Dean *et al.*, 2009 ▸; Sija *et al.*, 2014 ▸; Crisponi *et al.*, 2013 ▸).

### 3.2. Structural analysis of [Zn(A1)_2_(H_2_O)_2_] (1)   

**Figure 2:**
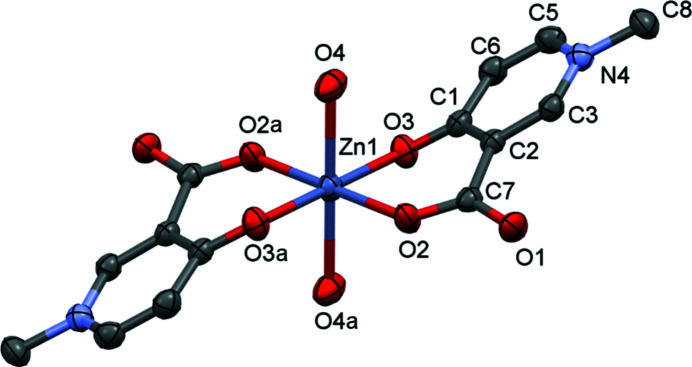
The molecular structure of [Zn(A1)_2_(H_2_O)_2_], **1**, with displacement ellipsoids drawn at the 50% probability level. H atoms have been omitted for clarity. For atom labels. suffix a = 1 − *x*, −*y*, 2 − *z*.

The single-crystal X-ray diffraction (SXRD) study showed that [Zn(A1)_2_(H_2_O)_2_] (**1**) crystallizes in the triclinic space group *P* 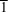. The asymmetric unit consists of half of the com­plex (half a metal ion, one anionic A1 ligand and one axially coordinated water molecule), as the Zn1 ion is positioned on an inversion centre (Fig. 2 ▸). Zn1 is six-coordinated, exhibiting a distorted octahedral geometry. The pyri­dine-ring plane deviates significantly from the coordination plane, as the dihedral angle between the planes generated by the coordinating atoms O2/Zn1/O3 and the pyri­dine ring is 23.59 (5)° (Table S1 in the supporting information), while the pyri­dine-ring planes are parallel owing to the centrosymmetrical arrangement. The obtained Zn—O distances in the coordination sphere agree with the usual distance of 2.1 ± 0.1 Å obtained from the CSD for the Zn—O bond length (Table 2 ▸). The two ligands coordinate to the metal ion in a *trans* arrangement (their carboxyl­ate groups are in opposite positions with respect to the equatorial plane). This *trans* coordination geometry of the ligands was found previously in the corresponding Cu^II^ com­plex. Ligand A1 coordinates to Cu^II^ with the two ligands in a *trans* arrangement, [Cu_2_(A1)_4_]·4H_2_O, and the noncoordinated carboxyl­ate O atom binds to a neighbouring Cu^II^ centre forming a *syn*–*anti* carboxyl­ate bridge in an equatorial–axial coordination mode, resulting in a cyclic dimer structure (Fig. S1 in the supporting information; May *et al.*, 2019 ▸). The geometry of the Zn^II^ com­plex of A1 is close to octahedral, while that of Cu^II^ is square pyramidal (Fig. S1 in the supporting information). The equatorial Cu—O2 and Cu—O3 bond lengths were found to be significantly shorter [1.931 (3) and 1.924 (3) Å, respectively] than the axial bond [2.614 (3) Å] in the crystal. In contrast, for **1**, the equatorial Zn—O2 and Zn—O3 distances [2.0249 (15) and 2.0701 (15) Å, respectively] are much closer to that of the axial Zn—O4 bond distance [2.1794 (19) Å]. The distances and angles measured between the atoms of the coordination sphere in the corresponding Zn^II^ and Cu^II^ com­plexes are collected in Table 2 ▸ and Table S2 in the supporting information. The packing arrangements of all measured crystals, viewed from the three crystallographic directions, are collected in Fig. S9 in the supporting information.

**Figure 3:**
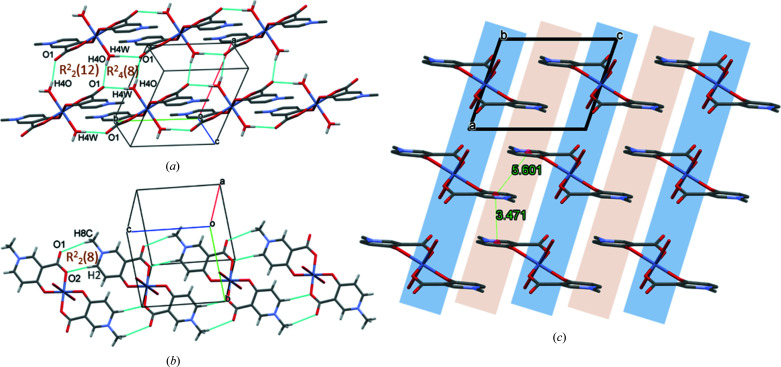
Packing arrangements in **1**, showing the main (*a*) O—H⋯O and (*b*) C—H⋯O intermolecular interactions, and (*c*) the alternating hydro­philic (blue) and hydro­phobic (grey) layers, viewed from the crystallographic *b* direction. Ring–ring; distances shown in (*c*) are in Å.

In **1**, the main secondary interaction is between the axially coordinated water H atoms and the carboxyl­ate O atom of an adjacent molecule (O4—H4O⋯O1^i^). It is repeated by the symmetry centres; thus, two hydrogen-bonded rings are formed, depicted as graph sets *R* 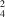
(8) and *R* 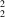
(12), respectively (Etter *et al.*, 1990 ▸) (Fig. 3 ▸ *a*). This strong intermolecular interaction arranges the molecules into a 2D sheet in the *ab* crystallographic plane. These sheets are connected by methyl and ring protons to adjacent carboxyl­ate O atoms, forming weak C8—H8*C*⋯O1^iii^ and C2—H2⋯O2^iii^ interactions (Fig. 3 ▸ *b*) in the *cb* plane. Short ring–ring interactions between parallel pyri­dine rings, with a distance of 3.4715 (16) Å, increase the stability of the lattice. The crystal contains alternating hydro­phobic and hydro­philic layers repeated in the crystallographic *c* direction (Fig. 3 ▸ *c*). Inter­atomic distances and angles of some selected secondary interactions are collected in Table 3 ▸.

### 3.3. Structural analysis of [Zn(A2)_2_(H_2_O)] (2)   

**Figure 4:**
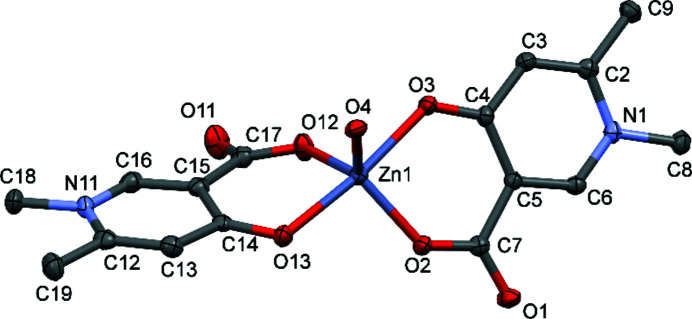
The molecular structure of [Zn(A2)_2_(H_2_O)], **2**, with displacement ellipsoids drawn at the 50% probability level. H atoms have been omitted for clarity.

The Zn^II^ crystal of A2, *i.e.* **2**, was colourless and block-shaped in the triclinic space group *P* 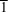 (the same as **1**). In **2**, however, the whole com­plex is in the asymmetric unit, not only half as in **1**, and the inversion centre is positioned between two adjacent molecules. The two ligands coordinate asymmetrically and this is manifested in different dihedral angles between the coordination plane and the pyri­dine ring planes. The first ligand lies almost in the coordination plane, as the angle between the O2/Zn1/O3 plane and the pyri­dine-ring plane is 1.88 (8)°, while for the second ligand, this angle is 22.95 (8)°. This also means that the two pyri­dine rings are closer to perpendicular than to planar geometry, and the angle between the two ring planes is 60.72 (11)° (see Table S1 in the supporting information). The two ligands coordinate in mutually *trans* positions, although the equatorial plane is highly distorted, resulting in an almost trigonal bipyramidal geometry (Fig. 4 ▸). The Zn^II^—O(carboxyl­ate) distances Zn1—O2 and Zn1—O12 are significantly shorter [1.9619 (17) and 1.9729 (17) Å, respectively] than the Zn^II^—O(oxide) bonds Zn1—O3 and Zn1—O13 [2.0799 (17) and 2.0454 (17) Å, respectively; see Table 2 ▸]. At the same time, the Zn—O bond to the aqua ligand has almost the same length [2.004 (2) Å] as those to the O2 and O12 donor groups, so that the trigonal bipyramidal geometry is supported. The O2—Zn1—O4 angle is 123.57 (8)° and the O2—Zn1—O12 angle is 113.47 (7)°, which are also close to the angle of 120° expected for a trigonal bipyramidal com­plex. In order to decide whether the geometry of the coordination centre is trigonal bipyramidal or square pyramidal, the τ_5_ (originally just τ) parameter was introduced by Addison *et al.* (1984 ▸). This parameter is calculated with the equation τ_5_ = (β − α)/60, where β > α are the two largest valence angles of the coordination centre. When τ_5_ is close to 0, the geometry is similar to square pyramidal, while if τ_5_ is close to 1, the geometry is similar to trigonal bipyramidal. In **2**, the τ_5_ value is 0.837, confirming the trigonal bipyramidal geometry. As a consequence of this conformation, the two ligands turn out of the equatorial plane so that the dihedral angle between the pyri­dine-ring planes of the two ligands is 60.72 (11)°.

In com­parison, the Cu^II^ com­plex with the A2 ligand displays a square-pyramidal geometry with axial bonding of the neighbouring carboxyl­ate O atom in a *syn*–*anti* coordination mode, resulting in a one-dimensional (1D) polymer chain (Fig. S2 in the supporting information; May *et al.*, 2019 ▸). The formation of 1D polymer chains was not unexpected because the methyl groups introduced into the pyri­dine ring inhibit the formation of a cyclic dimer similar to that obtained in the case of [Cu_2_(A1)_4_]·4H_2_O (Fig. S1). Selected distances and angles measured in the coordination sphere of the Zn^II^ and Cu^II^ com­plexes with ligand A2 are collected in Table 2 ▸ and Table S2 in the supporting information, respectively.

**Figure 5:**
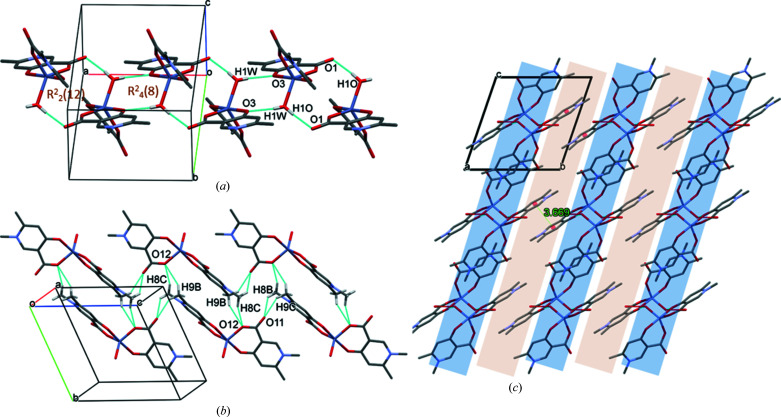
The packing arrangements in **2**, showing the main (*a*) O—H⋯O and (*b*) C—H⋯O intermolecular interactions, and (*c*) the alternating hydro­philic (blue) and hydro­phobic (grey) layers, viewed from the crystallographic *a* direction. The ring–ring; distance shown in (*c*) is in Å.

In **2**, the complex molecules are arranged in 1D columns along the crystallographic *c* axis, organized by O4—H1O⋯O1^iv^ [graph-set descriptor *R* 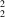
(12) and O4—H1*W*⋯O3^v^ [*R* 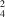
(8)] interactions (Fig. 5*a* ▸) around an inversion centre placed in the middle of each ring of intermolecular inter­actions. There are inter-column C—H⋯O interactions between the methyl protons and carboxylate O atoms, *i.e.* C8—H8*C*⋯O12^vii^, C8—H8*B*⋯O11^vi^, C9—H9*B*⋯O12^vii^ and C9—H9*C*⋯O11^vi^ (Fig. 5*b* ▸). Face-to-face π–π interactions, with a distance of 3.669 (12) Å, are present between the pyridine rings. The crystal contains alternating hydrophobic and hydrophilic layers repeated in the crystallographic *a* direction (Fig. 5*c* ▸), with more distortion than in **1**.

### 3.4. Structural analysis of [Zn(A3)_2_(H_2_O)]·2H_2_O (3)   

**Figure 6:**
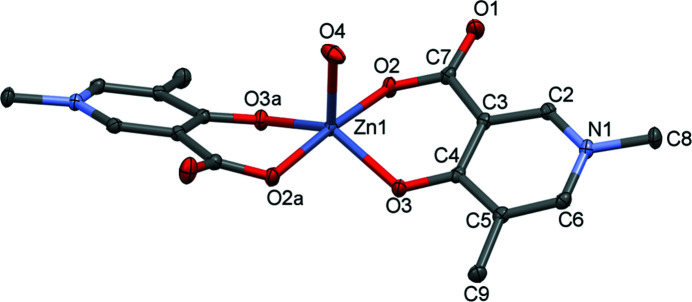
The molecular structure of [Zn(A3)_2_(H_2_O)]·2H_2_O, **3**, with displacement ellipsoids drawn at the 50% probability level. H atoms and water molecules of crystallization have been omitted for clarity. For atom labels, suffix a = −*x*, *y*, 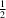 − *z*.

The SXRD study shows that **3** crystallizes in the monoclinic space group *C*2/*c*. The asymmetric unit consists of half of the com­plex (half a metal ion with half of the axially coordinated water molecule, one A3 ligand and one water molecule of crystallization), as the Zn1—O4 bond lies on a twofold axis (Fig. 6 ▸). The dihedral angle between the planes generated by the coordinating atoms (O2/Zn1/O3) and pyri­dine ring is 28.26 (5)° for both ligands, and the angle between the two pyri­dine-ring planes is 41.32 (9)° (Table S1 in the supporting information). Zn1 is five-coordinated, exhibiting a geometry between square pyramidal and trigonal bipyramidal. The τ_5_ parameter was calculated to be 0.592, which is less than in the case of **2**, but is still closer to trigonal bipyramidal geometry than to square pyramidal. No com­parison is possible for the com­plex formed by A3 with Cu^II^ as the latter has not be crystallized thus far. The conformations of the two trigonal bipyramidal structures (**2** and **3**) differ considerably (Fig. S10 in the supporting information). The Zn—O2, Zn—O3 and Zn—O4 bond lengths are almost equal in this com­plex [1.9993 (10)–2.0282 (12) Å; Table 2 ▸], so that the water O atom has a similar binding strength to the ligand O-donor atoms. The two ligands are rotated out of the equatorial plane, but the angle between the two pyri­dine-ring planes is smaller [41.32 (9) Å] than in **2** [60.72 (11) Å]. Bond lengths and angles measured between the atoms of the coordination sphere are collected in Table 2 ▸.

**Figure 7:**
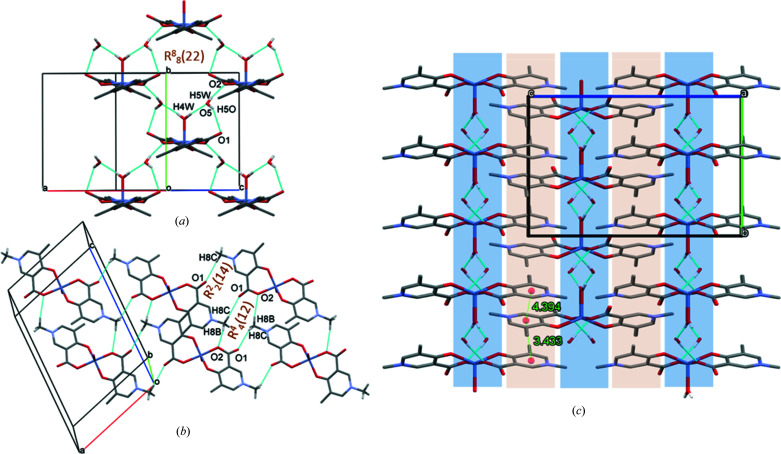
The packing arrangements in **3**, showing the main (*a*) O—H⋯O and (*b*) C—H⋯O intermolecular interactions, and (*c*) the alternating hydro­philic (blue) and hydro­phobic (grey) layers, viewed from the crystallographic *a* direction. Ring–ring distances are shown in Å.

There are similarities in the packing arrangements of com­plexes **2** and **3** (com­pare Fig. 6 ▸ *b* with Fig. 7 ▸ *b*), as the carboxyl­ate O atoms bind to >N^+^—Me protons in both structures, *via* C8—H8*C*⋯O12^vii^ and C8—H8*B*⋯O11^vi^ hydrogen bonds in **2**, and C8—H8*B*⋯O2^x^ and C8—H8*C*⋯O1^v^ hydrogen bonds in **3** (Table 3 ▸). While the protons of the axially coordinated water molecule are connected directly to the carboxyl­ate O atom of the neighbouring com­plex in the complexes of A1 and A2, in the complex with A3, the water protons and the neighbouring carboxyl­ate O atoms are connected through a water molecule of crystallization as a bridge between the com­plex molecules below and above each other (Fig. 7 ▸ *a*). The main hydrogen-bond interactions, O4—H4*W*⋯O5^viii^, O5—H5O⋯O1^ix^ and O5—H5*W*⋯O2, connect four complex molecules in a ring by the graph set *R* 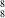
(22), organized by twofold and twofold screw axes, and intersected by a glide plane but lacking inversion symmetry. These are further connected, forming a 2D sheet in the crystallographic *ab* plane; data are shown in Table 3 ▸. Carboxyl­ate O atoms are connected with the >N^+^—Me group protons of an adjacent com­plex in a neighbouring plane by C8—H8*B*⋯O2^x^, forming an *R* 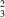
(14) ring, and by C8—H8*C*⋯O1^v^, forming an *R* 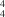
(12) ring (Fig. 7 ▸ *b*). The shortest pyri­dine–pyri­dine ring distance measured between the centres of gravity of two rings is 3.4325 (9) Å. The alternating hydro­phobic and hydro­philic sheets can be recognized even in this structure (Fig. 7 ▸ *c*).

### 3.5. Structural analysis of [Zn_2_(B1)_4_.(H_2_O)_2_]·4H_2_O (4)   

**Figure 8:**
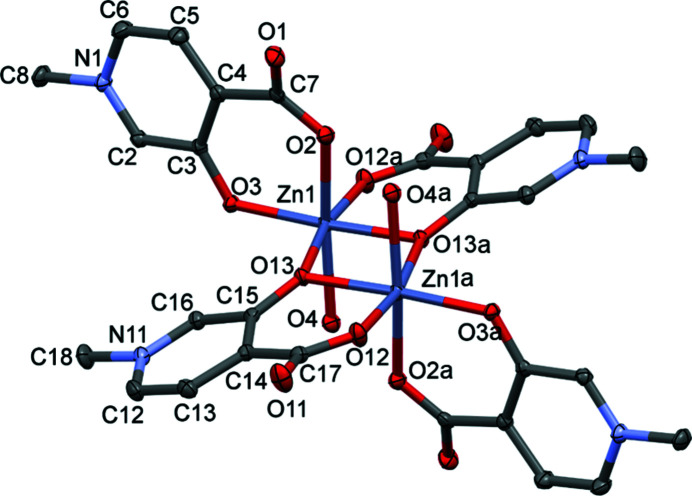
The molecular structure of [Zn_2_(B1)_4_(H_2_O)_2_]·4H_2_O, **4**, with displacement ellipsoids drawn at the 50% probability level. H atoms and water molecules of crystallization have been omitted for clarity. For atom labels, suffix a = −*x*, 2 − *y*, 1 − *z*.

In ligand B1, the positions of the oxide and carboxyl­ate groups on the pyridine ring are interchanged, which alters significantly the electron distribution of the O-donor atoms. In the case of the Cu^II^ com­plex, *i.e.* [Cu(B1)_2_(H_2_O)]·3H_2_O, this results in the coordination of the two ligands in a *cis* arrangement (Fig. S3 in the supporting information) instead of the *trans* arrangement that was observed for the Zn^II^ and Cu^II^ com­plexes of A1. The Cu^II^ com­plex is five-coordinated in a square-pyramidal geometry, with a water molecule coordinated in the axial position. In the case of Zn^II^, however, the com­plex of B1 resulted in a dimeric structure. This was a surprising result as the formation of dimeric (dinuclear com­plex) of HPCs with Zn^II^ has not been reported to occur in the solution state (Sija *et al.*, 2014 ▸). The dimeric complex crystallizes in the triclinic space group *P* 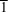 and has two coordinated water molecules and four additional water molecules of crystallization. One Zn^2+^ ion, two ligands, one coordinated axially, and two solvent water molecules form the asymmetric unit (Fig. 8 ▸), and the other half of the dimeric complex is formed repeating this part through an inversion centre positioned between the two Zn1 ions. Shorter Zn—O bond lengths are found in the six-membered chelate rings (Zn1—O3, Zn1—O2, Zn1—O12 and Zn1—O13), while longer bond lengths are found for the water Zn1—O4 and the bridging oxide Zn1—O13 bonds (Table 2 ▸).

**Figure 9:**
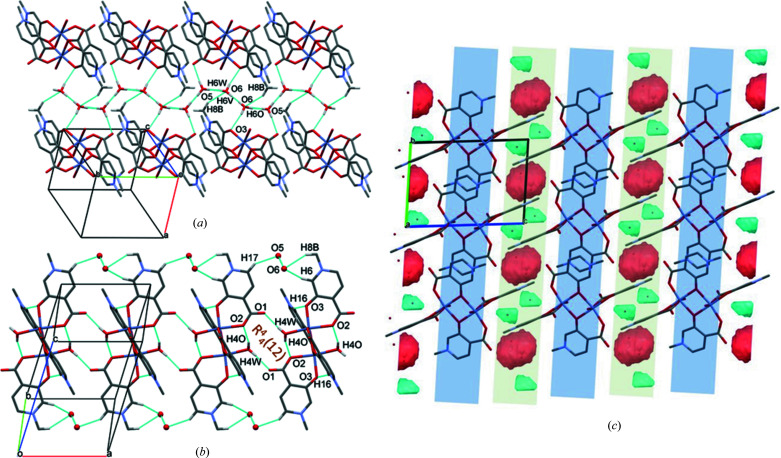
The packing arrangements in **4**, showing the main (*a*) O—H⋯O and (*b*) C—H⋯O intermolecular interactions, and (*c*) layers, viewed from the crystallographic *a* direction. Hydro­phobic layers (blue) found in **1**–**3** alternate with layers (green) which contain water molecules of crystallization (red and green voids) together with the aromatic rings.

The packing of the molecules in **4** is dominated by O—H⋯O hydrogen bonds with the participation of the axially coordinated water protons and the water molecules of crystallization. The intramolecular O4—H4O⋯O2^xi^ interaction between the axially coordinated water and the carboxyl­ate O atom stabilizes the binuclear com­plex, while the other coordinated water proton connects two dimeric units together [graph set *R* 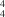
(12)], thus forming a chain of com­plex molecules in the crystallographic *a* direction. The O5 and O6 water molecules are located in channels in the crystallographic *b* direction and are involved in hydrogen bonds as hydrogen-bond donors in three different directions. They are connected to the acceptor O3 and O11 atoms of the ligands, respectively. Furthermore, the direction of the hydrogen bonds alternates in the columns formed by the water molecules of crystallization; thus, the protons appear between the two O atoms connected alternately to one of them and so both protons could be found in difference Fourier maps. These protons (H5*V*/H5*W* and H6*V*/H6*W*) were refined with an occupancy of 0.5. The water molecules of crystallization take part in further C—H⋯O interactions as acceptors with the >N^+^—Me protons of adjacent ligands (Fig. 9 ▸). Selected hydrogen-bond parameters of **4** are collected in Table 3 ▸.

The alternating packing arrangement observed in the crystal structures of **1**–**3** is modified in **4** as a result of the exchange of the positions of the carboxylate and oxide groups (Fig. 9 ▸ *c*). The former hydro­phobic layer is com­pleted, with the zigzag chain of connected water molecules of crystallization separating neighbouring com­plexes.

### 3.6. Comparison of the supramolecular interactions by Hirshfeld surface analysis   

**Figure 10:**
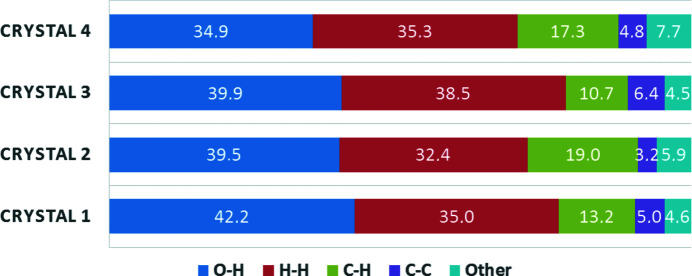
The relative contributions (%) of the various intermolecular contacts to the Hirshfeld surface area in **1**–**4** (for further details, see Fig. S11 in the supporting information).

The Hirshfeld surfaces of the investigated molecules in **1**–**4** were calculated in order to com­pare the supramolecular interactions (Fig. S11–S13 in the supporting information). The relative contributions of the main intermolecular contacts O⋯H/H⋯O, H⋯H, C⋯H/H⋯C and C⋯C are shown in Fig. 10 ▸. The ratio of the O⋯H/H⋯O contacts is the highest in **1**, presumably because there are two axial water molecules in this com­plex, while the others have only one. There is one more methyl group in ligands A2 and A3 com­pared to A1, which should increase the H⋯H contacts in the crystal, and this can be seen in the case of **3**. However, in **2**, the relative contribution of H⋯H contacts to the Hirshfeld surfaces has decreased. At the same time, the percentage of C⋯H contacts increases in **2** com­pared to **1**, which means that neighbouring ligands are packed in such a way that the methyl protons are closer to the C atoms of the pyri­dine ring than to each other. The largest contribution of the C⋯C contact to the Hirshfeld surface can be seen in **3**, which also has the shortest ring–ring distance. In **4**, the Hirshfeld surface was calculated for the dimer unit. Despite the presence of the four water molecules of crystallization, the percentage of O⋯H/H⋯O contacts is lowest here, likely because the water molecules are mainly connected to each other in a channel in the crystal lattice.

### 3.7. Comparison of the coordination spheres in 1–4   

Two of the investigated bis-ligand Zn^II^ com­plexes contain a six-coordinated metal ion (with ligand A1 and B1) and the other two com­plexes exhibit five-coordination (with ligands A2 and A3). Although the two donor groups of the ligands could have resulted in a tetrahedral geometry with four-coordination, the coordination spheres were com­pleted in all cases by one or two water molecules, resulting in five- or six-coordination geometries instead.

**Figure 11:**
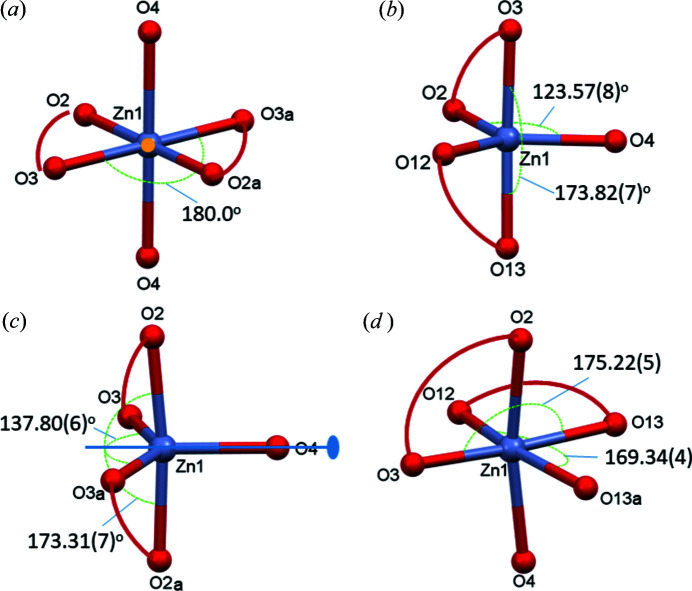
Comparison of the coordination spheres of Zn^II^ in (*a*) **1** (for atom labels, suffix *a* = 1 − *x*, *y*, 2 − *z*), (*b*) **2**, (*c*) **3** (for atom labels, suffix *a* = −*x*, *y*, 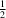 − *z*) and (*d*) **4** (for atom labels, suffix *a* = −*x*, 2 − *y*, 1 − *z*). The largest valence angles are shown.

The coordination spheres in the investigated crystals are com­pared in Fig. 11 ▸. A highly symmetrical octahedral geometry with two axial water molecules and an inversion centre coinciding with the Zn^II^ position was detected in the com­plex of ligand A1 (crystal **1**) (Fig. 11 ▸ *a*). A less symmetrical octahedron is seen in the dimer of B1 (crystal **4**), with one axial water molecule and an equatorial coordination of the neighbouring ligand O13*a* atom (Fig. 11 ▸ *b*). The bis-ligand Zn^II^ com­plexes of A2 and A3 display a five-coordinated trigonal bipyramidal geometry in **2** and **3**, with the coordination of one water molecule (Figs. 11 ▸ *c* and 11*d*). The differences between the structures of **2** and **3** are as follows: (i) in **3**, a twofold rotation axes coincides with the Zn1—O4 bond, while this symmetry element is missing in **2**, and (ii) in **2**, the ligand carboxyl­ate O atoms (O2 and O12) coordinate equatorially to the metal ion, while it is the oxide O atom (O3) in the case of **3**. The calculated τ_5_ values (0.837 and 0597, respectively) show a more symmetrical trigonal bipyramidal geometry for **2** than for **3**. Fig. S10 (see in the supporting information) shows an overlay of the crystal structures of **2** and **3**.

**Figure 12:**
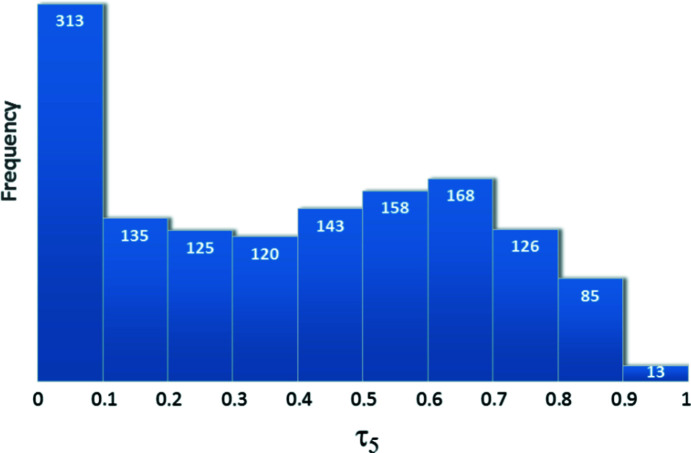
Histogram showing the τ_5_ values for 5O-coordinated Zn^II^ com­plexes deposited in the CSD.

In order to investigate the occurrences of the different geometries among five-coordinated Zn^II^ com­plexes, conformational data were collected from the CSD. Up until early 2020, 3176 structures of solely oxygen-coordinated Zn^II^ com­plexes had been deposited with the coordination of exactly four O-atom donors (4O-coordination), 1284 with 5O-coordination and 2906 with 6O-coordination. This distribution shows that Zn^II^ has a preference to form tetragonal and hexagonal com­plexes, although five-coordination is also seen, but with slightly fewer occurrences. In the case of five-coordination, the 1284 entries contained 1629 Zn^II^ com­plexes with 1386 different structures left after filtering out entries with identical structures. The τ_5_ value for each was calculated to establish the distribution of the occurrences of square-pyramidal and trigonal bipyramidal structures among the 5O-coordinated Zn^II^ com­plexes. The histogram obtained from the distribution of the occurrences of the τ_5_ values is shown in Fig. 12 ▸. The highest occurrence can be observed with low τ_5_ values (τ_5_ = 0–0.1), which belongs to square-pyramidal geometry. The higher τ_5_ values show a more even distribution until τ_5_ < 0.8, with a maximum around 0.65. With τ_5_ above 0.8, the number of occurrences is significantly decreased, so that only a few structures have closely symmetrical trigonal bipyramidal geometry. It can be deduced that **2** and **3** are atypical structures, and the τ_5_ value of 0.837 in the case of ligand A2 corresponds to a rather unusual Zn^II^ com­plex geometry. As a com­parison, τ_5_ values were also calculated for the 5O-coordinated Cu^II^ com­plexes, and it was found that the incidence of trigonal bipyramid geometry here is even lower, as 97% (τ_5_ < 0.5) of the structures have square-pyramidal geometry (Fig. S14 in the supporting information). In our analogous Cu^II^ com­plexes of ligands A1, A2 and B1, only square-pyramidal geometry was found.

The preference to form bis-ligand com­plexes in a *trans* orientation remained for ligands A1, confirming that this arrangement depends on the electronic distribution of the ligands and not on the crystal packing. In the case of Cu^II^, the geometry is primarily determined by the crystal field, resulting in square-pyramidal geometry being energetically more favourable.

The splitting of the *d*-orbitals, as a consequence of the Jahn–Teller effect, appears to be the greatest driving force in the design of the com­plexes, resulting in a less tight fit of the Cu^II^ com­plexes; the gaps between the com­plexes are then filled with water molecules of crystallization (three or four water molecules per com­plex; see Figs. S15–S17 in the supporting information). It can be concluded from the coordination analysis that the coordination geometry of the Zn^II^ com­plexes is more flexible, and the coordination sphere accommodates better to the neighbouring com­plexes to form a tight fit in the crystal lattice. This is reflected in the water content of the relevant Cu^II^ and Zn^II^ crystals. While in [Cu_2_(A1)_4_]·4H_2_O and {[Cu(A2)_2_]·3H_2_O}_*n*_, the water of crystallization is 11.5 and 11.4% of the unit-cell volume, respectively (Figs. S15 and S16 in the supporting information), the Zn^II^ analogues **1** and **2** do not contain water of crystallization. In the case of B1, the formed dimer fixes the geometry around the Zn^II^ ions and the water content is similar to and as high as in the Cu^II^ analogue (7.2 and 5.6%, respectively; see Figs. S17 and S18 in the supporting information).

We note that the com­plex crystals have been isolated from aqueous solutions containing a twofold ligand excess, and other ligand-to-metal concentration ratios or solvents have not been tested, so that the formation of other crystal forms or coordination geometries under different con­ditions cannot be excluded. Based on our previous solution speciation study (Sija *et al.*, 2014 ▸), we may expect that by dissolving any of the investigated [Zn*L*_2_] crystals in aqueous solution, it would partially dissociate and a mixture of mono- and bis-ligand com­plexes would be obtained. The formation of square-py­rami­dal or octahedral Zn^II^ com­plexes is more likely in solution than trigonal bipyramidal com­plexes, which are stabilized by secondary interactions in the crystals, and the formation of the *cis* isomer cannot be excluded either. Solution equilibrium studies did not show dimer formation with Zn^II^, but with Cu^II^, dimer formation could be detected in the frozen solution by electron spin resonance (ESR) spectroscopy (May *et al.*, 2019 ▸).

## 4. Conclusions   

The single-crystal structures of bis-ligand Zn^II^ com­plexes of four *O*,*O*′-donor hy­droxy­pyri­dine­carboxyl­ate ligands have been determined. Despite their identical bidentate coordination modes, various geometries have been obtained. The most common geometry in the case of Zn^II^ with a *d* ^10^ electronic configuration would be tetragonal or octahedral, based on crystal field theory. Here, after crystallization water solution at pH ∼ 7, the formation of one octahedral com­plex with two axially coordinated water molecules, [Zn(A1)_2_(H_2_O)_2_] (**1**), two com­plexes with one axial water coordination and trigonal bipyramidal geometry, [Zn(A2)_2_(H_2_O)] (**2**) and [Zn(A3)_2_(H_2_O)]·2H_2_O (**3**), and a dimeric structure with an oxide O-atom bridge, [Zn_2_(B1)_4_(H_2_O)_2_]·4H_2_O (**4**), was established. The structural variety is probably not only due to the electronic differences between the ligands, but also to the adaptation of the coordination geometry to the close crystal packing to maximize the attractive interaction between ligands and to create a tight fit in the crystal. There is a common packing pattern containing alternating hydro­philic and hydro­phobic layers irrespective of the substitution, coordination and space group. This pattern can be broken only by the exchange of the positions of the oxide and carboxylate groups.

Comparing the Zn^II^ com­plexes with the Cu^II^ analogues, we conclude that, due to the different number of electrons (*d* ^9^ for Cu^II^), square-pyramidal geometry with a longer axial bond is preferred in the bis-ligand copper com­plexes. In these com­plexes of A1 and A2, the axial donor atom is a neighbouring carboxyl­ate O atom, while in B1 it is a water O atom. In these Cu^II^ analogues, a large number of molecules of water of crystallization was found, so that the geometry of the com­plex appears to be strongly fixed by regulation of crystal field and the voids between the molecules are filled with solvent molecules. In this case, the packing of the com­plexes does not induce any effect on the geometry of the com­plex, in contrast to that observed for zinc com­plexes. However, we cannot exclude the formation of other possible geometries of these com­plexes in other crystal forms by the use of different crystallization con­ditions and solvents.

A comprehensive CSD study of Zn com­plexes coordinated with a different number of O atoms has been performed. According to the CSD, we conclude that trigonal bipyramidal geometry is rather uncommon for 5O-coordinated zinc com­plexes, and with τ_5_ > 0.8, the geometry obtained in **2** is quite rare.

## Supplementary Material

Crystal structure: contains datablock(s) global, 1, 2, 3, 4. DOI: 10.1107/S2052520621000299/lo5086sup1.cif

Structure factors: contains datablock(s) 1. DOI: 10.1107/S2052520621000299/lo50861sup2.hkl

Structure factors: contains datablock(s) 2. DOI: 10.1107/S2052520621000299/lo50862sup3.hkl

Structure factors: contains datablock(s) 3. DOI: 10.1107/S2052520621000299/lo50863sup4.hkl

Structure factors: contains datablock(s) 4. DOI: 10.1107/S2052520621000299/lo50864sup5.hkl

Additional Tables and Figures. DOI: 10.1107/S2052520621000299/lo5086sup6.pdf

CCDC references: 2009100, 2009101, 2009102, 2009103

## Figures and Tables

**Table 1:** Experimental details

	**1**	**2**	**3**	**4**
Crystal data				
Chemical formula	[Zn(C_7_H_6_NO_3_)_2_(H_2_O)_2_]	[Zn(C_8_H_8_NO_3_)_2_(H_2_O)]	[Zn(C_8_H_8_NO_3_)_2_(H_2_O)]·2H_2_O	[Zn_2_(C_7_H_6_NO_3_)_4_(H_2_O)_2_]·4H_2_O
*M*_r_	405.66	415.71	451.72	847.35
Crystal system, space group	Triclinic, *P*\overline{1}	Triclinic, *P*\overline{1}	Monoclinic, *C*2/*c*	Triclinic, *P*\overline{1}
Temperature (K)	293	138	103	103
*a*, *b*, *c* (Å)	7.2924 (5), 7.4450 (5), 8.0936 (6)	8.2959 (3), 10.1989 (4), 10.3466 (4)	10.8962 (4), 10.3334 (3), 16.6916 (7)	7.9237 (5), 8.5740 (5), 12.3954 (8)
α, β, γ (°)	97.966 (7), 103.385 (7), 115.659 (5)	70.187 (5), 82.585 (6), 79.659 (6)	90, 108.435 (1), 90	84.248 (2), 74.706 (2), 85.908 (2)
*V* (Å^3^)	370.76 (5)	808.01 (6)	1782.94 (11)	807.32 (9)
*Z*	1	2	4	1
Radiation type	Cu *K*α	Cu *K*α	Mo *K*α	Mo *K*α
μ (mm^−1^)	2.79	2.53	1.43	1.58
Crystal size (mm)	0.45 × 0.40 × 0.30	0.50 × 0.20 × 0.10	0.50 × 0.50 × 0.30	0.50 × 0.20 × 0.20

Data collection				
Diffractometer	Rigaku R-AXIS RAPID	Rigaku R-AXIS RAPID	Rigaku R-AXIS RAPID	Rigaku R-AXIS RAPID
Absorption correction	Numerical (*NUMABS*; Higashi, 2011 ▸)	Numerical (*NUMABS*; Higashi, 2011 ▸)	Numerical (*NUMABS*; Higashi, 2002 ▸)	Numerical (*NUMABS*; Higashi, 2002 ▸)
*T*_min_, *T*_max_	0.682, 0.828	0.637, 0.894	0.396, 0.765	0.429, 0.777
No. of measured, independent and observed [*I* > 2σ(*I*)] reflections	5106, 1225, 1209	11 343, 2856, 2715	35 961, 2046, 2020	27 896, 3680, 3521
*R*_int_	0.029	0.045	0.046	0.035
(sin θ/λ)_max_ (Å^−1^)	0.602	0.602	0.649	0.649

Refinement				
*R*[*F* ^2^ > 2σ(*F* ^2^)], *wR*(*F* ^2^), *S*	0.029, 0.075, 1.16	0.034, 0.088, 1.07	0.028, 0.075, 1.19	0.024, 0.063, 1.10
No. of reflections	1225	2856	2046	3680
No. of parameters	116	247	142	263
No. of restraints	0	0	0	6
H-atom treatment	H-atom parameters constrained	H atoms treated by a mixture of independent and constrained refinement	H atoms treated by a mixture of independent and constrained refinement	H atoms treated by a mixture of independent and constrained refinement
Δρ_max_, Δρ_min_ (e Å^−3^)	0.34, −0.20	0.53, −0.36	0.58, −0.37	0.50, −0.33

Computer programs: *RAPID-AUTO* (Rigaku, 2015 ▸) for **1** and **2**, *CrystalClear* (Rigaku/MSC, 2008 ▸) for **3** and **4**, *SIR2014* (Burla *et al.*, 2015 ▸), *SHELXT* (Sheldrick, 2015*a* ▸), *SHELXL2014* (Sheldrick, 2015*b* ▸), *Mercury* (Macrae *et al.*, 2020 ▸), *PLATON* (Spek, 2020 ▸), *WinGX* (Farrugia, 2012 ▸) and *publCIF* (Westrip, 2010 ▸).

**Table 2:** Selected interatomic distances (Å) and angles (°) in **1**–**4**

	**1**	**2**	**3**	**4**
Zn1—O2	2.0249 (15)	1.9619 (17)	2.0282 (12)	2.1198 (12)
Zn1—O3	2.0701 (15)	2.0799 (17)	1.9993 (10)	1.9998 (11)
Zn1—O12	–	1.9729 (17)	–	2.0302 (12)
Zn1—O13	–	2.0454 (17)	–	2.0563 (11)
Zn1—O13a^i^	–	–	–	2.1613 (11)
Zn1—O4_ax_	2.1794 (19)	2.0042 (18)	2.0046 (19)	2.1478 (12)
				
O2—Zn1—O3	88.05 (6)	88.70 (7)	88.05 (4)	89.59 (4)
O2—Zn1—O2	180	–	173.31 (7)	–
O3 —Zn1—O3	180	–	137.80 (6)	–
O2—Zn1—O4_ax_	89.61 (7)	123.57 (8)	93.34 (3)	169.23 (5)
O3 —Zn1—O4_ax_	87.39 (7)	88.70 (7)	111.10 (3)	96.94 (5)
O2—Zn1—O12	–	113.47 (7)	–	–
O3—Zn1—O13	–	173.82 (7)	–	175.22 (5)
O12—Zn—O13	–	89.62 (7)	–	169.34 (4)

Symmetry code: (i) −*x*, 2 − *y*, 1 − *z*.

**Table 3:** Hydrogen-bond geometry (Å, °) for **1**–**4**

*D*—H⋯*A*	*D*—H	H⋯*A*	*D*⋯*A*	*D*—H⋯*A*
Crystal **1**				
O4—H4O⋯O1^i^	0.82	2.01	2.814 (3)	167
O4—H4*W*⋯O1^ii^	0.84	1.92	2.752 (3)	174
C2—H2⋯O2^iii^	0.93	2.34	3.205 (3)	155
C8—H8*C*⋯O1^iii^	0.96	2.66	3.354 (3)	129

Crystal **2**				
O4—H1O⋯O1^iv^	0.84 (4)	1.87 (4)	2.708 (3)	175 (4)
O4—H1*W*⋯O3^v^	0.74 (3)	1.93 (3)	2.668 (2)	174 (4)
C8—H8*B*⋯O11^vi^	0.96	2.55	3.296 (4)	135
C8—H8*C*⋯O12^vii^	0.96	2.37	3.286 (3)	159
C9—H9*B*⋯O12^vii^	0.96	2.55	3.434 (4)	152
C9—H9*C*⋯O11^vi^	0.96	2.38	3.140 (3)	136

Crystal **3**				
O4—H4*W*⋯O5^viii^	0.79 (3)	1.84 (3)	2.626 (2)	175 (3)
O5—H5O⋯O1^ix^	0.79 (3)	2.45 (3)	3.139 (2)	147 (3)
O5—H5*W*⋯O2	0.78 (3)	1.97 (3)	2.745 (2)	173 (3)
C8—H8*B*⋯O2^x^	0.98	2.52	3.496 (2)	174
C8—H8*C*⋯O1^v^	0.98	2.48	3.330 (2)	145

Crystal **4**				
O4—H4O⋯O2^xi^	0.84 (1)	2.00 (1)	2.823 (2)	164 (2)
O4—H4*W*⋯O1^xii^	0.83 (2)	1.88 (2)	2.710 (2)	175 (2)
O5—H5O⋯O11^xiii^	0.84 (2)	1.93 (2)	2.761 (2)	174 (2)
O5—H5*V*⋯O6	0.73 (6)	2.04 (6)	2.765 (2)	179 (2)
O5—H5*W*⋯O5^xiv^	0.84 (4)	1.86 (4)	2.700 (2)	178 (4)
O6—H6O⋯O3^ii^	0.83 (2)	2.03 (2)	2.853 (2)	168 (2)
O6—H6*V*⋯O6^xv^	0.81 (5)	1.95 (5)	2.750 (2)	178 (7)
O6—H6*W*⋯O5	0.84 (2)	1.94 (2)	2.765 (2)	170 (5)
C2—H2⋯O6^xiv^	0.95	2.53	3.230 (2)	131
C8—H8*B*⋯O6^xiv^	0.98	2.48	3.383 (2)	152
C6—H6⋯O5^xvi^	0.95	2.47	3.179 (2)	132

Symmetry codes: (i) −*x*, −*y*, −*z* + 2; (ii) *x*, *y* − 1, *z*; (iii) *x*, *y*, *z* − 1; (iv) −*x* + 2, −*y* + 1, −*z* + 1; (v) −*x* + 1, −*y* + 1, −*z* + 1; (vi) *x*, *y*, *z* + 1; (vii) −*x* + 1, −*y* + 2, −*z* + 1; (viii) −*x* + 1 \over 2, *y* + 1 \over 2, −*z* + 1 \over 2; (ix) −*x* + 1 \over 2, *y* − 1 \over 2, −*z* + 1 \over 2; (x) *x*, −*y* + 1, *z* + 1 \over 2; (xi) −*x*, −*y* + 2, −*z* + 1; (xii) *x* + 1, *y*, *z*; (xiii) −*x*, −*y* + 1, −*z* + 1; (xiv) −*x* + 1, −*y* + 1, −*z*; (xv) −*x* + 1, −*y*, −*z*; (xvi) −*x*, −*y* + 1, −*z*.
