# Supplementary material for: Crystal structures of zinc(II) com­plexes with β-hy­droxy­pyri­dine­carboxyl­ate ligands: examples of structure-directing effects used in inorganic crystal engineering
Source: Acta Crystallogr B Struct Sci Cryst Eng Mater. 2021 Feb 27;77(Pt 2):193–204. doi: 10.1107/S2052520621000299 (PMC8040096; doi:10.1107/S2052520621000299)
Supplement: Supplementary file 6 [file b-77-00193-sup6.pdf]

Acta Cryst B

STRUCTURAL SCIENCE
CRYSTAL ENGINEERING
MATERIALS

Volume 77 (2021)

Supporting information for article:

Crystal structures of zinc(II) complexes with β-hydroxypyridine-carboxylate ligands: examples for structure-directing effects used in inorganic crystal engineering

Nóra Veronika May, Kevin Nys, H. Y. Vincent Ching, Laura Bereczki, Tamás Holczbauer, Valerio B. Di Marco and Petra Bombicz

Table S1 Selected Interatomic Distances (Å) and Dihedral angles (deg) for the ligands A1 – A3 and B1 in crystals (1)-(4)

	Crystal **1**	Crystal **2**	Crystal **3**	Crystal **4**
Distances (Å)				
O1-C7	1.255(3)	1.244(3)	1.232(2)	1.248(2)
O2-C7	1.256(3)	1.270(3)	1.288(2)	1.268(2)
O3-C4/C3	1.268(3)	1.278(3)	1.283(2)	1.302(2)
N1-C2	1.366(3)	1.375(3)	1.364(2)	1.335(2)
C2-C3	1.344(4)	1.363(4)	1.363(2)	1.415(2)
C3-C4	1.428(3)	1.420(3)	1.444(2)	1.425(2)
C4-C5	1.450(3)	1.437(3)	1.434(2)	1.395(2)
C5-C6	1.363(3)	1.366(3)	1.382(2)	1.378(2)
C6-N1	1.346(3)	1.349(3)	1.340(2)	1.351(2)
N1-C8	1.468(3)	1.475(3)	1.477(2)	1.482(2)
O11-C17	-	1.233(3)	-	1.247(2)
O12-C17	-	1.278(3)	-	1.262(2)
O13-C14/C13	-	1.272(3)	-	1.303(3)
N11-C12	-	1.374(3)	-	1.341(2)
C12-C13	-	1.363(4)	-	1.419(2)
C14-C15	-	1.438(3)	-	1.395(2)
C15-C16	-	1.364(3)	-	1.375(2)
C16-N11	-	1.345(3)	-	1.352(2)
N11-C18	-	1.473(3)	-	1.484(2)
Dihedral angles (deg)				
P(Zn1-O2-O3) – P(pyr1)[a]	23.5	1.9	28.3	13.6
P(Zn1-O12-O13) – P(pyr2)[b]	-	22.9	-	22.7
P(pyr1)-P(pyr2)[c]	0.0	60.7	41.3	76.1

[a]Dihedral angle between the planes generated by Zn1-O2-O3 atoms and pyridine ring N1-C2-C3-C4-C5-C6. [b]Dihedral angle between the planes generated by Zn1-O12-O13 atoms and pyridine ring N11-C12-C13-C14-C15-C16. [c] Dihedral angels between the planes generated by the two pyridine rings

Table S2 Selected Interatomic Distances (Å) and Angles (deg) for the analogous copper(II) complexes of ligands **A1**, **A2** and **B1**

	$[Cu_2(\mathbf{A1})_4].4H_2O$	$[Cu(\mathbf{A2})_2]_n.3H_2O$	$[Cu(\mathbf{B1})_2(H_2O)].3H_2O$
**Cu1-O2**	1.931(3)	1.937(2)	1.957(1)
**Cu1-O3**	1.924(3)	1.949(2)	1.935(1)
**Cu1-O1**	1.937(3)	1.919(2)	1.926(1)
**Cu1-O13**	1.921(3)	1.921(2)	1.916(1)
**Cu1-O$_{ax}$**	2.614(3)	2.388(2)	2.234(2)
**O2-Cu1-O3**	93.1(1)	90.83(9)	90.36(6)
**O13-Cu1-O12**	92.7(1)	93.61(9)	94.23(6)
**O3-Cu1-O13**	172.3(1)	165.0(1)	86.24(6)
**O12-Cu1-O2**	178.6(1)	172.9(1)	87.22(6)
**O3-Cu1-O12**	88.2(1)	86.55(9)	172.57(6)
**O2-Cu1-O13**	86.1(1)	87.21(9)	164.38(7)

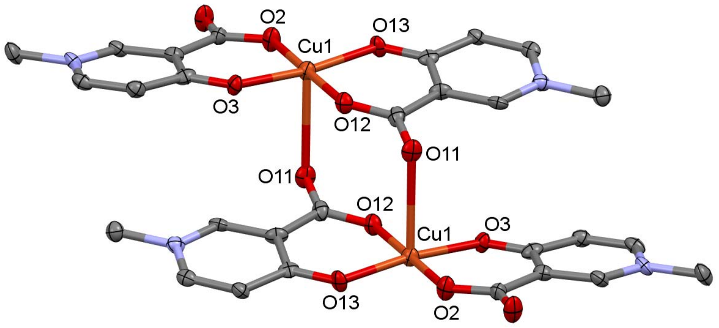

Figure S1 ORTEP view of crystal $[Cu_2(\mathbf{A1})_4].4H_2O$ (Ref. code NOHVEO) thermal displacement ellipsoids drawn at 50% probability level. Hydrogen atoms and waters of crystallization are omitted for clarity.

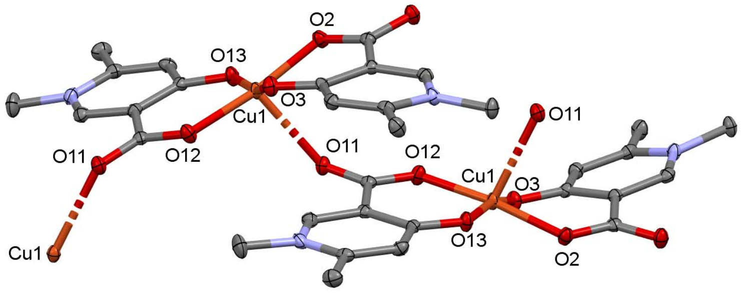

ORTEP view of crystal $[Cu(\mathbf{A2})_2]_n.3H_2O$ (Ref. code NOHVIS) coordination polymer with thermal displacement ellipsoids drawn at the 50% probability level. Hydrogen atoms and lattice water molecules are omitted for clarity.

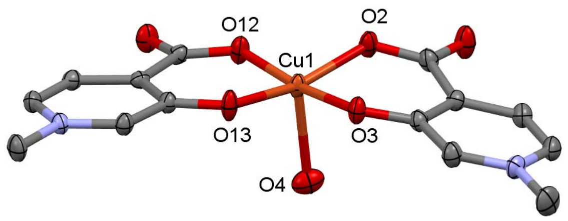

ORTEP view of crystal $[Cu(\mathbf{B1})_2(H_2O)].3H_2O$ (Ref. code NOHVAK) with thermal displacement ellipsoids drawn at the 50% probability level. Hydrogen atoms and waters of crystallisation are omitted for clarity.

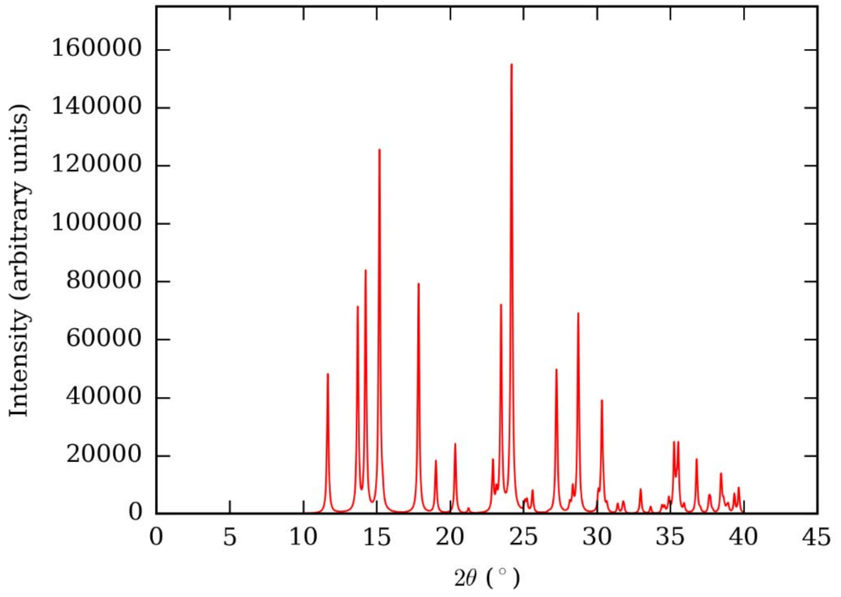

Figure S2 Powder profile of crystal 1 simulated by PLATON (wavelength = 1.54187 Å).

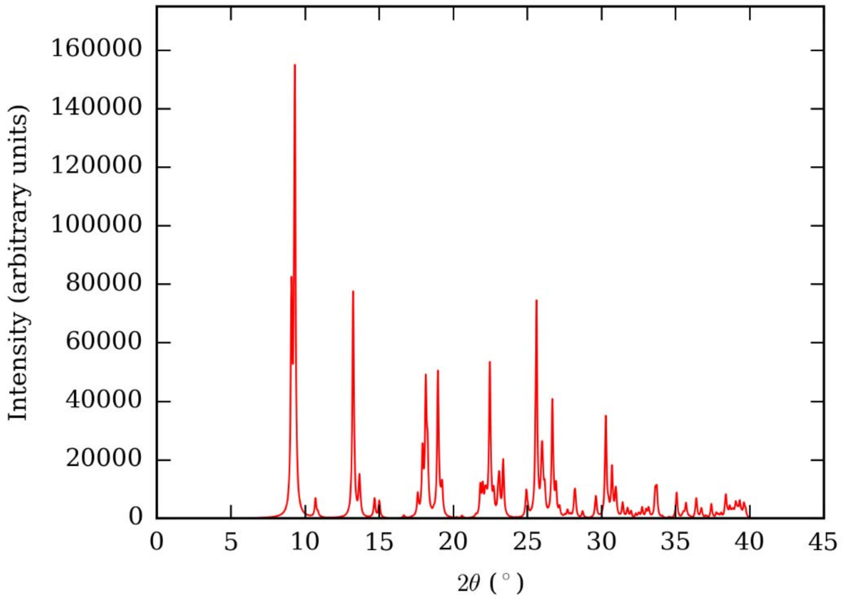

Figure S3 Powder profile of crystal 2 simulated by PLATON (wavelength = 1.54187 Å)

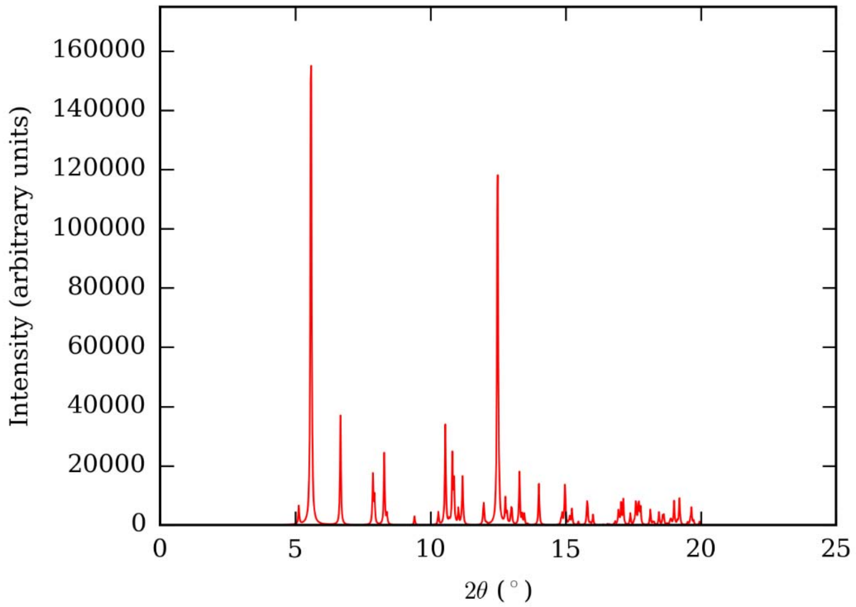

Figure S4 Powder profile of crystal 3 simulated by PLATON (wavelength = 1.54187 Å)

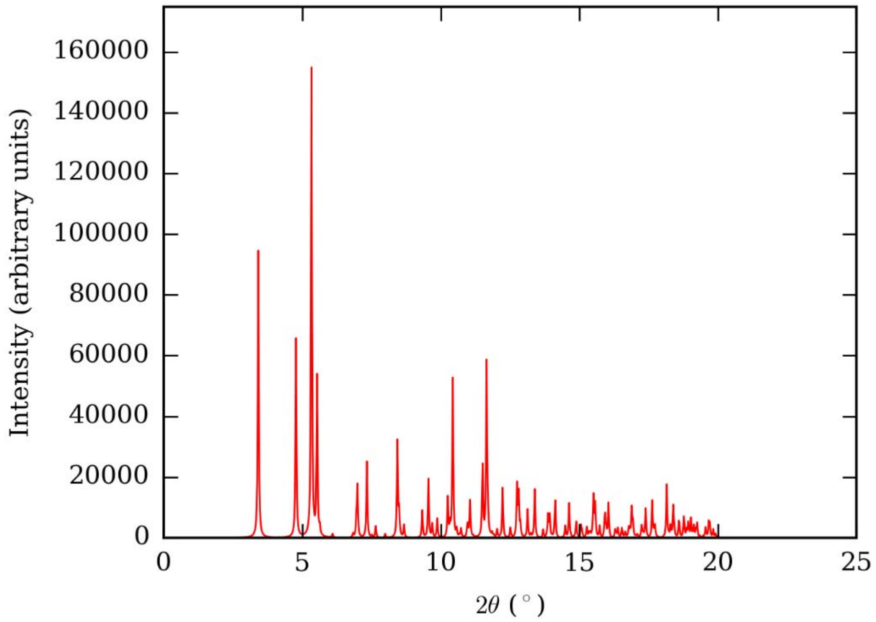

Figure S5 Powder profile of crystal 4 simulated by PLATON (wavelength = 1.54187 Å)

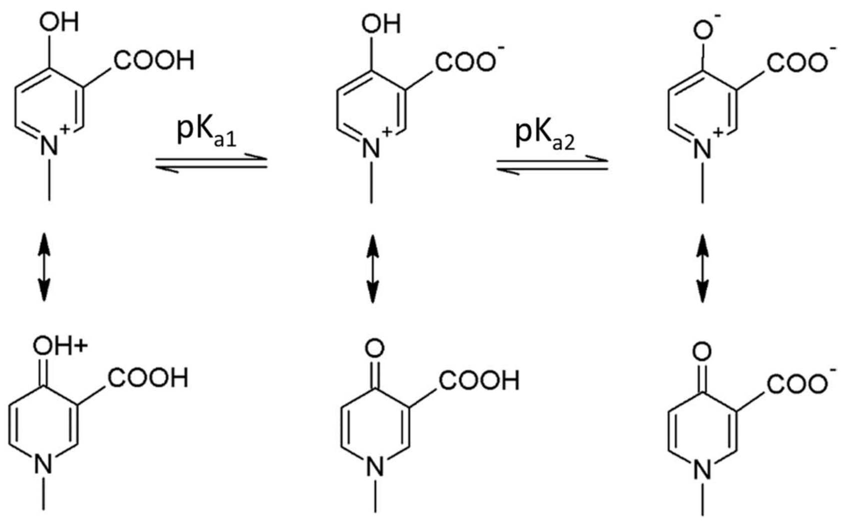

A1H$_2^+$ **A1H** **A1$^-$**

Figure S6 Deprotonation steps for ligand A1 indicating possible enol-oxo tautomer forms

(a)

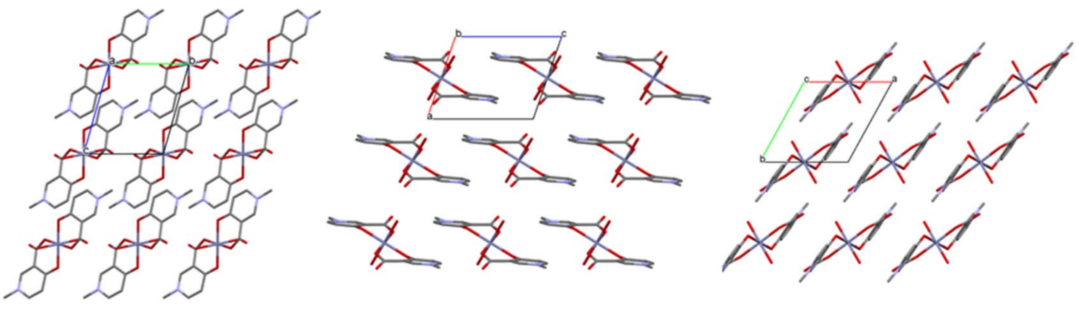

(b)

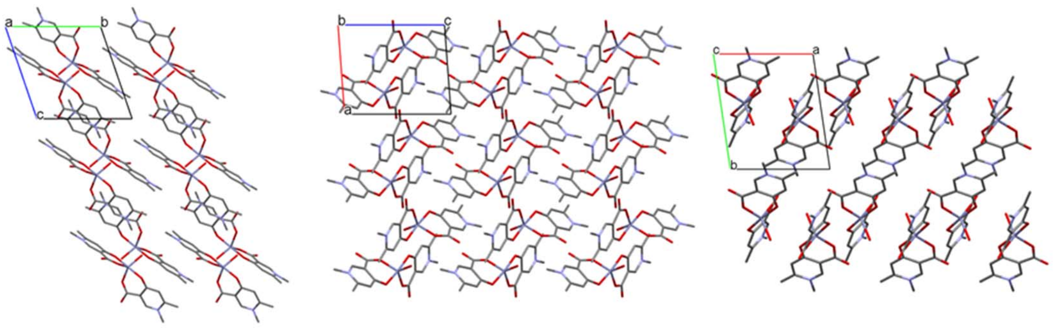

(c)

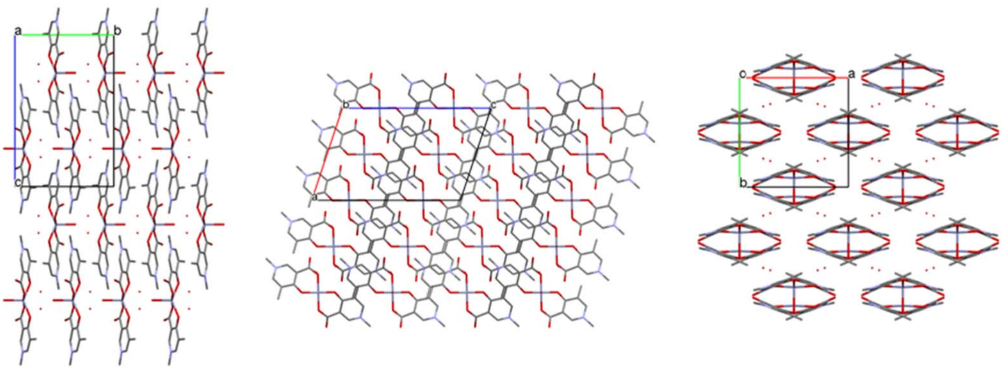

(d)

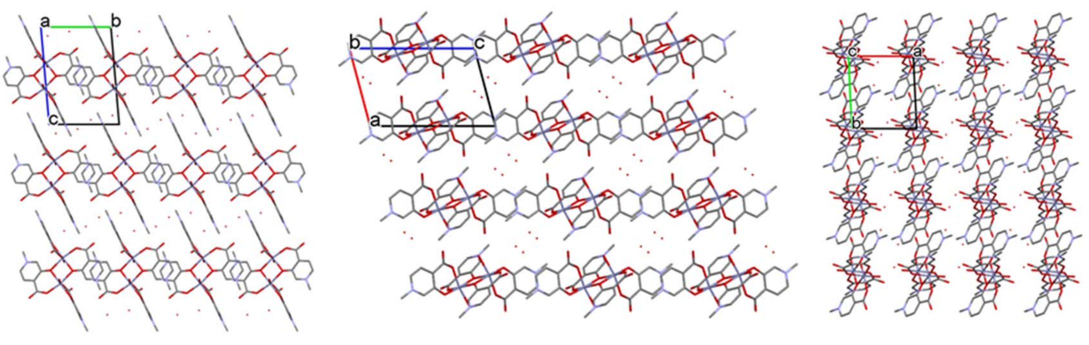

Figure S7 Crystal packing viewed from the crystallographic directions ‘a’, ‘b’ and ‘c’ in (a) crystal **1**, (b) crystal **2**, (c) crystal **3** and (d) crystal **4**

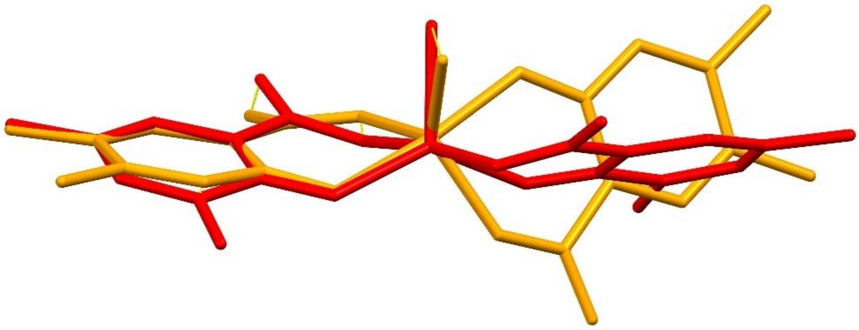

Figure S8 Overlay of the two trigonal bi-pyramidal Zn complexes **2** (orange) and **3** (red).

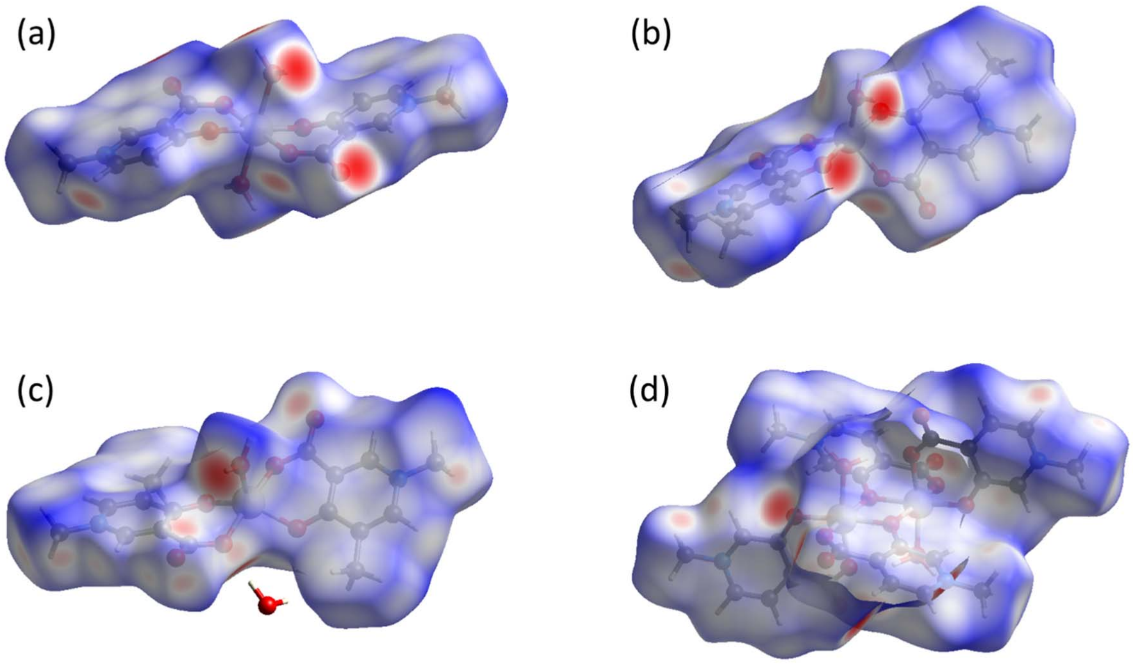

Figure S9 Hirshfeld surface, showing the close contact points in red, calculated for the zinc(II) complexes in (a) crystal 1, (b) crystal 2, (c) crystal 3 and (d) crystal 4

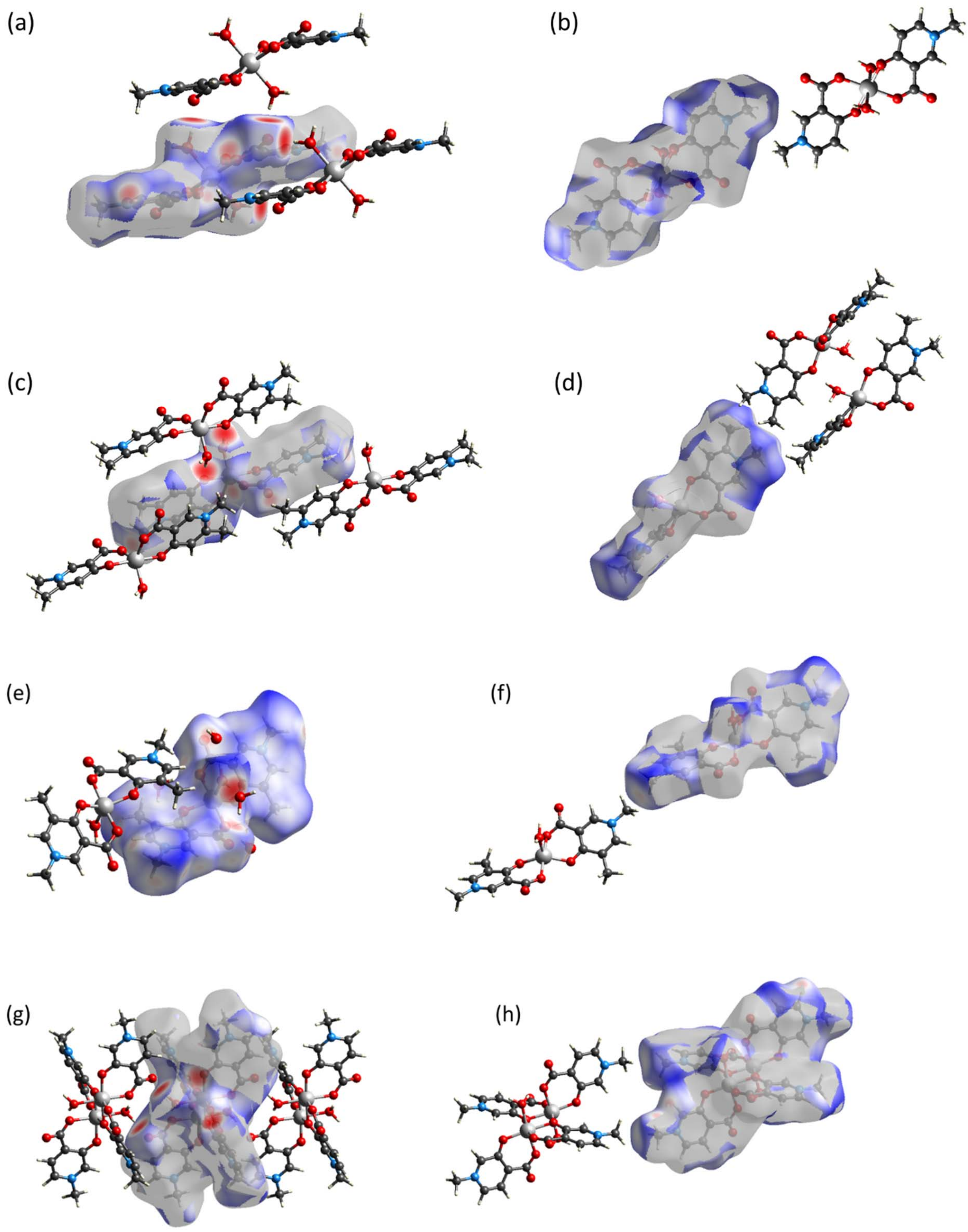

Figure S10 Hirshfeld surface, showing the close contact points in red, between O…H atoms (a,c,e,g) and H…H atoms (b,d,f,g), for the zinc(II) complexes in crystal 1 (a and b), crystal 2 (c and d), crystal 3 (e and f) and crystal 4 (g and h). The molecular surface coloured in gray does not take part in the corresponding interactions.

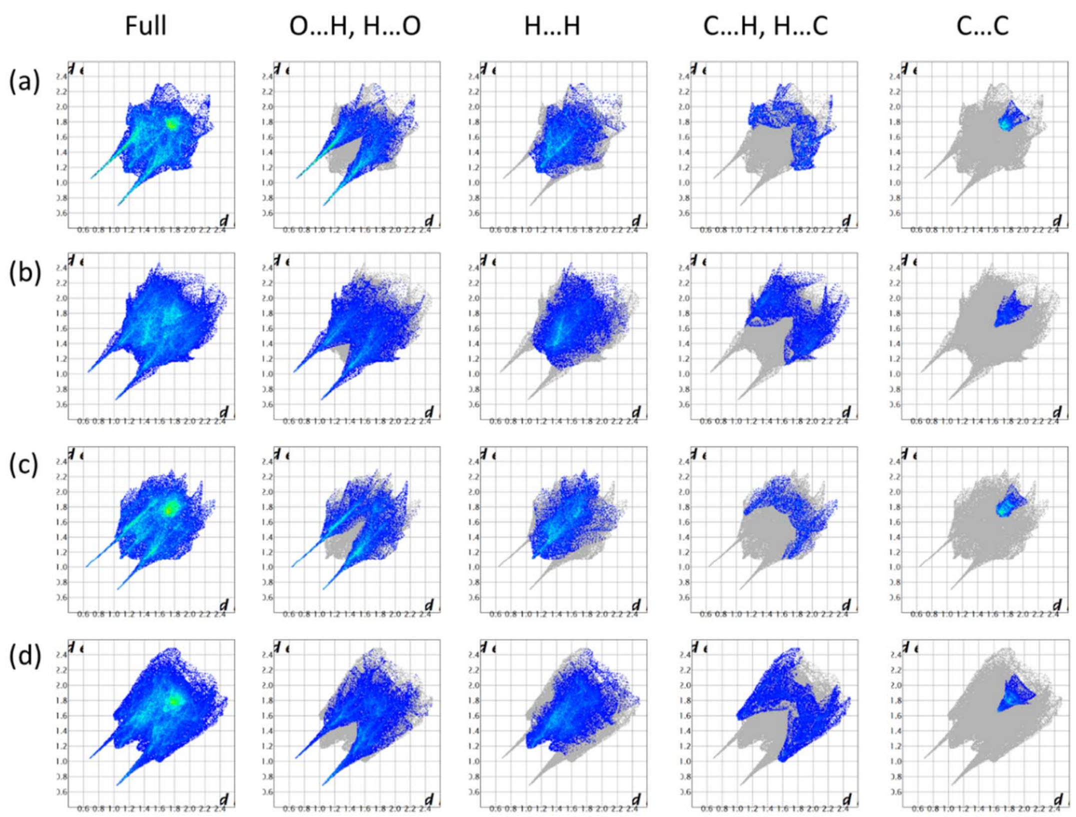

Figure S11 Two dimensional fingerprint plots showing all the interactions (Full), and highlighting the O…H,H…O, H…H, C…H,H…C and C…C contacts, in (a) crystal 1, (b) crystal 2, (c) crystal 3 and (d) crystal 4

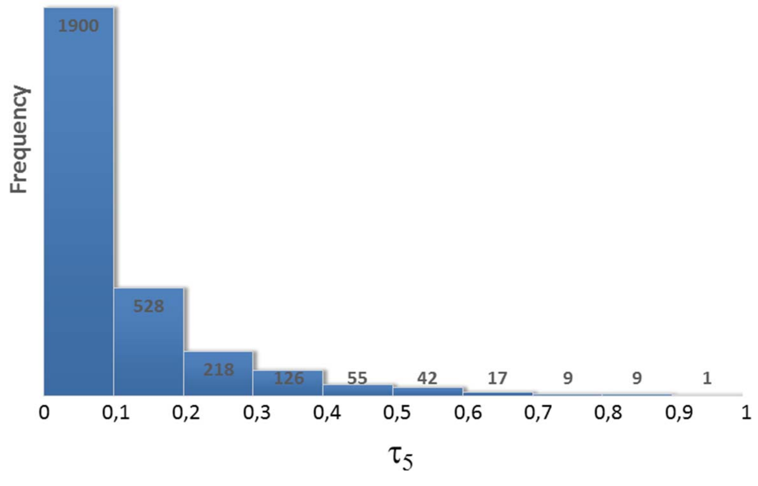

Figure S12 Histogram showing the τ_5 values for 5O –coordinated copper complexes deposited in the CSD. The histogram shows the τ_5 values calculated for 2905 structures obtained in 2457 entries for 5O-coordinated Cu(II) complexes, after filtering out the identical structures. For 4O-coordination 1659 and for 6O-coordination 2184 entries have been found in the CSD.

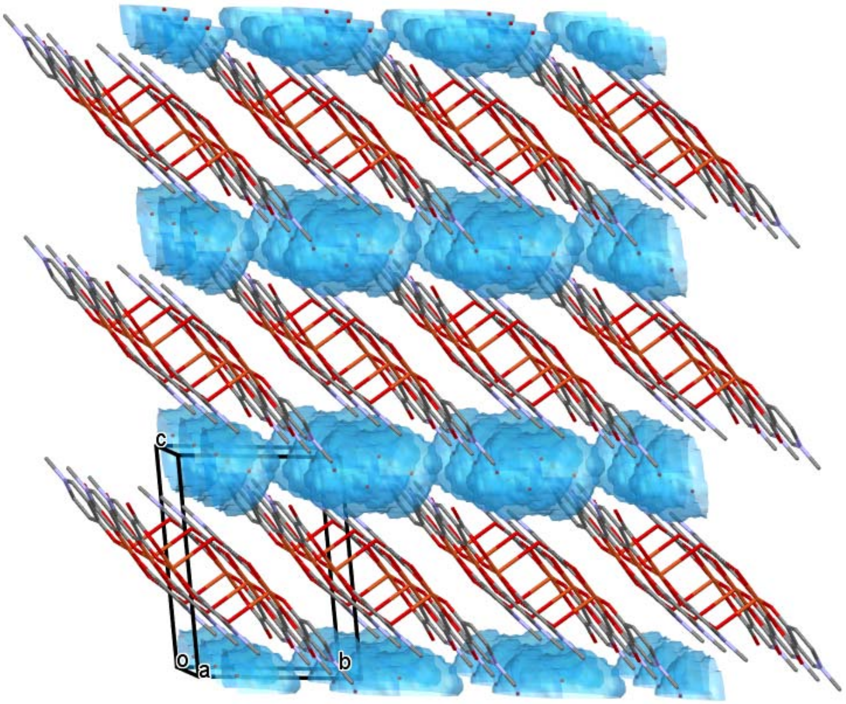

Figure S13 Packing arrangements of crystal [$Cu_2(A1)_4$].$4H_2O$ (Ref. code NOHVEO) showing voids of 84.4 Å^3 (11.5% of unit cell) where water of crystallization is placed (calculated by Mercury, Hydrate Analyser)

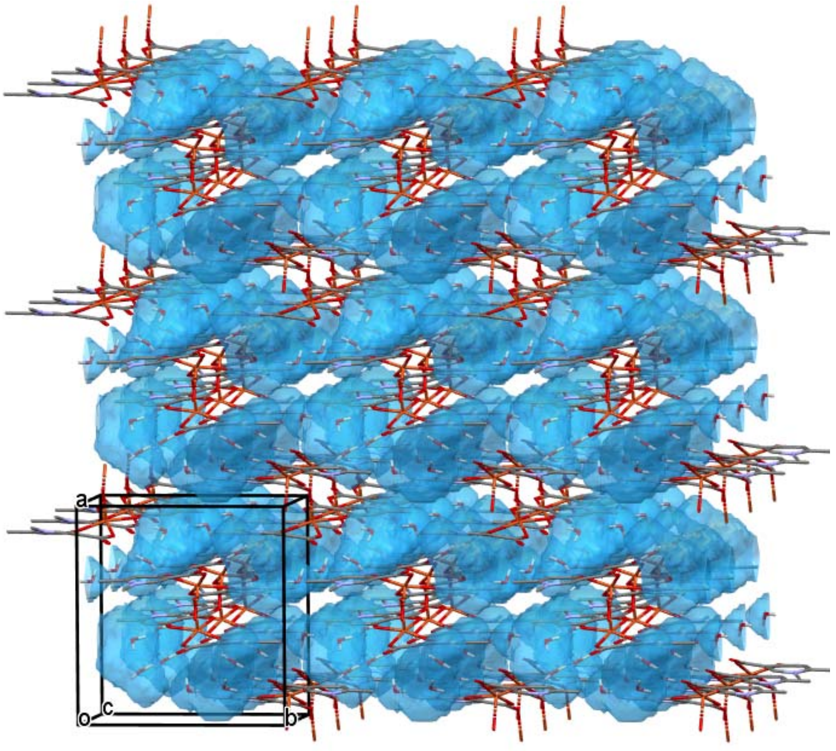

Figure S14 Packing arrangement in crystal [$Cu(A2)_2$]$_n$.$3H_2O$ (Ref. code NOHVIS) with channels created by the water molecules. The volume of the voids is 204.7 Å^3 wich is 11,4 % of the unit cell volume (calculated by Mercury, Hydrate Analyser).

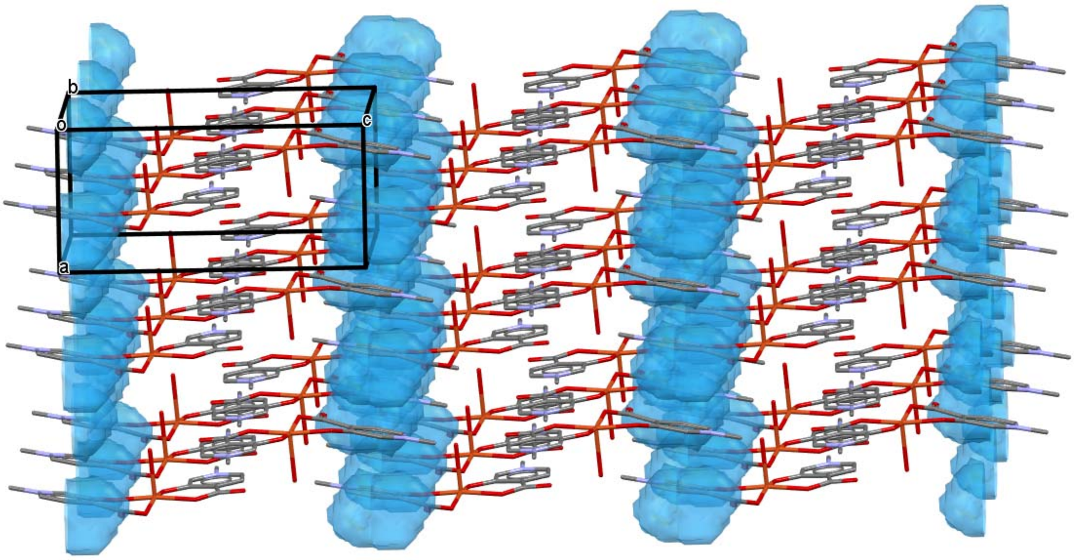

Figure S15 Packing arrangement of crystal [Cu(B1)$_2$(H$_2$O)].3H$_2$O (Ref. code NOHVAK) showing voids of 48,9 Å^3 (5.6% of the unit cell) where water of crystallization is placed (calculated by Mercury, Hydrate Analyser).

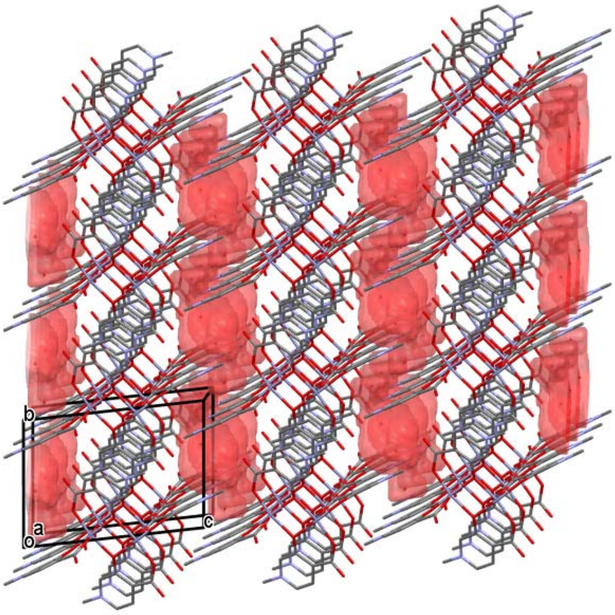

Figure S16 Packing arrangement in crystal [Zn$_2$(**B1**)$_4$(H$_2$O)].4H$_2$O (**4**) showing voids of 58,4 Å^3 (7.2% of the unit cell) where water of crystallization is placed (calculated by Mercury).
